# Supplementary material for: Prediction of Asbestos-Related Diseases (ARDs) and Chrysotile Asbestos Exposure Concentrations in Asbestos-Cement (AC) Manufacturing Factories in Zimbabwe
Source: Int J Environ Res Public Health. 2022 Dec 21;20(1):58. doi: 10.3390/ijerph20010058 (PMC9819734; doi:10.3390/ijerph20010058)
Supplement: Supplementary file 1 [file ijerph-20-00058-s001.zip › ijerph-2040850-supplementary.pdf]

Supplementary Material

Prediction of Asbestos Related Diseases (ARDs) and chrysotile asbestos exposure concentrations in asbestos-cement (AC) manufacturing factories in Zimbabwe.

Benjamin Mutetwa [1,*], Dingani Moyo [1,2,3] and Derk Brouwer [1]

[1] School of Public Health, Faculty of Health Sciences, University of the Witwatersrand, 2193, Johannesburg, South Africa
[2] Faculty of Medicine and Health Sciences, Midland State University, Gweru 054, Zimbabwe
[3] Department of Community Medicine, Faculty of Medicine, National University of Science and Technology, Bulawayo 029, Zimbabwe
* Correspondence: bjmtetwa@yahoo.com; Tel.: +263-773-429-838

1. Table S1: Cumulative exposure on lung cancer risk by job, time period, exposure duration of 1, 10, 20 and 25 years: Harare factory
2. Table S2: Cumulative exposure on lung cancer risk by job, time period, exposure duration of 1, 10, 20 and 25 years: Bulawayo factory
3. Table S3: Cumulative exposure on mesothelioma risk by job, time period, exposure duration of 1, 10, 20 and 25 years: Harare factory
4. Table S4: Cumulative exposure on mesothelioma risk by job, time period, exposure duration of 1, 10, 20 and 25 years: Bulawayo factory
5. Table S5: Cumulative exposure on gastrointestinal cancer risk by job, time period, exposure duration of 1, 10, 20 and 25 years: Harare factory
6. Table S6: Cumulative exposure on gastrointestinal cancer risk by job, time period, exposure duration of 1, 10, 20 and 25 years: Bulawayo factory
7. Table S7: Overall summary estimates of cancer mortality cases by factory, job and duration of exposure of 1 year.
8. Table S8: Overall summary estimates of cancer mortality cases by factory, job and duration of exposure of 10 years.
9. Table S9: Overall summary estimates of cancer mortality cases by factory, job and duration of exposure of 20 years.
10. Table 10: Overall summary estimates of cancer mortality cases by factory, job and duration of exposure of 25 years.
11. Table S11**:** Estimates of possible asbestosis incidence after 25 years of exposure
12. Table S12: Estimated number of workers working at various jobs in the chrysotile asbestos cement manufacturing factories

Table S1: Cumulative exposure on lung cancer risk by job, time period, exposure duration of 1, 10, 20 and 25 years: Harare factory

			Duration of exposure											
			**1 year**			**10 years**			**20 years**			**25 years**		
Job	Time period	Mean f/ml	CE f/ml-years	Cases per/10^5	% Incid	CE f/ml-years	Cases per/10^5	% Incid	CE f/ml-years	Cases per/10^5	% Incid	CE f/ml-years	Cases per/10^5	% Incid
Saw cutting operator	1996-2000	0.19	0.19	13.7	0.01	1.9	135	0.14	3.8	274	0.27	4.8	346	0.35
	2001-2008	0.13	0.13	9.4	0.009	1.3	94	0.09	2.6	187	0.19	3.3	238	0.24
	2009-2016	0.07	0.07	5.1	0.005	0.7	50	0.05	1.4	101	0.10	1.8	130	0.13
	2018-2020	0.10	0.10	7.2	0.007	1.0	72	0.07	2.0	144	0.14	2.5	180	0.18
Fettling table operator	1996-2000	0.12	0.12	8.6	0.009	1.2	86	0.09	2.4	173	0.17	3.0	216	0.22
	2001-2008	0.12	0.12	8.6	0.009	1.2	86	0.09	2.4	173	0.17	3.0	216	0.22
	2009-2016	-	-	-	-	-	-	-	-	-	-	-	-	-
	2018-2020	0.11	0.11	7.9	0.008	1.1	79	0.08	2.2	158	0.16	2.8	202	0.20
Moulded goods operator	1996-2000	0.11	0.11	7.9	0.008	1.1	79	0.08	2.2	158	0.16	2.8	202	0.20
	2001-2008	0.11	0.11	7.9	0.008	1.1	79	0.08	2.2	158	0.16	2.8	202	0.20
	2009-2016	0.05	0.05	3.6	0.004	0.5	36	0.04	1.0	72	0.07	1.3	94	0.09
	2018-2020	0.11	0.11	7.9	0.008	1.1	79	0.08	2.2	158	0.16	2.8	202	0.20
Kollergang operator	1996-2000	0.13	0.13	9.4	0.01	1.3	94	0.09	2.6	187	0.19	3.3	238	0.24
	2001-2008	0.12	0.12	8.6	0.009	1.2	86	0.09	2.4	173	0.17	3.0	216	0.22
	2009-2016	0.07	0.07	5.0	0.005	0.7	50	0.05	1.4	101	0.10	1.8	130	0.13
	2018-2020	0.12	0.12	8.6	0.007	1.2	86	0.09	2.4	173	0.17	3.0	216	0.22
Ground hard waste operator	1996-2000	0.16	0.16	11.5	0.01	1.6	115	0.12	3.2	230	0.23	3.3	238	0.24
	2001-2008	0.13	0.13	9.4	0.009	1.3	94	0.09	2.6	187	0.19	3.3	238	0.24
	2009-2016	0.07	0.07	5.0	0.005	0.7	50	0.05	1.4	101	0.07	1.8	130	0.13
	2018-2020	0.12	0.12	8.6	0.009	1.2	86	0.09	2.4	173	0.16	3.0	216	0.22
Laundry room operator	1996-2000	0.13	0.13	9.4	0.009	1.3	94	0.09	2.6	187	0.19	3.3	238	0.24
	2001-2008	0.13	0.13	9.4	0.009	1.3	94	0.09	2.6	187	0.19	3.3	238	0.24
	2009-2016	0.05	0.05	3.6	0.004	0.5	36	0.04	1.0	72	0.07	1.3	94	0.09
	2018-2020	0.11	0.11	7.9	0.008	1.1	79	0.08	2.2	158	0.15	2.8	202	0.20
Overall factory		0.11	0.11	7.9	0.008	1.1	79	0.08	2.2	158	0.16	2.8	202	0.20

% Incid – Percentage incidence; CE – Cumulative. Cases/10^5 – cancer mortality/morbidity cases per 100 000 workers exposed.

Table S2: Cumulative exposure on **lung cancer** risk by job, time period, exposure duration of 1, 10, 20 and 25 years: Bulawayo factory

			**Duration of exposure**											
			**1 year**			**10 years**			**20 years**			**25 years**		
Job	Time period	Mean f/ml	CE f/ml-years	Cases per/10^5	% Incid	CE f/ml-years	Cases per/10^5	% Incid	CE f/ml-years	Cases per/10^5	% Incid	CE f/ml-years	Cases per/10^5	% Incid
Saw cutting operator	1996-2000	0.17	0.17	12.2	0.01	1.7	122	0.12	3.4	245	0.25	4.3	310	0.30
	2001-2008	0.12	0.12	8.6	0.01	1.2	86	0.09	2.4	173	0.17	3.0	216	0.20
	2009-2016	0.06	0.06	4.3	0.005	0.6	36	0.04	1.2	86	0.09	1.5	108	0.11
	2018-2020	0.05	0.05	3.6	0.007	0.5	36	0.04	1.0	72	0.07	1.3	94	0.09
Fettling table operator	1996-2000	0.17	0.17	12.2	0.01	1.7	122	0.12	3.4	245	0.25	4.3	310	0.30
	2001-2008	0.12	0.12	8.6	0.009	1.2	86	0.09	2.4	173	0.17	3.0	216	0.22
	2009-2016	-	-	-	-	-	-	-	-	-	-	-	-	
	2018-2020	-	-	-	-	-	-	-	-	-	-	-	-	
Moulded goods operator	1996-2000	-	-	-	-	-	-	-	-	-	-	-	-	-
	2001-2008	-	-	-	-	-	-	-	-	-	-	-	-	-
	2009-2016	-	-	-	-	-	-	-	-	-	-	-	-	-
	2018-2020	-	-	-	-	-	-	-	-	-	-	-	-	-
Kollergang operator	1996-2000	0.14	0.14	10.1	0.01	1.4	101	0.10	2.8	202	0.20	3.5	252	0.25
	2001-2008	0.12	0.12	8.6	0.009	1.2	86	0.09	2.4	173	0.17	3.0	216	0.22
	2009-2016	0.07	0.07	5.0	0.005	0.7	50	0.05	1.4	101	0.10	1.8	130	0.13
	2018-2020	0.06	0.06	4.3	0.004	0.6	36	004	1.2	86	0.09	1.5	108	0.11
Ground hard waste operator	1996-2000	0.13	0.13	9.4	0.009	1.3	94	0.09	2.6	187	0.19	3.3	238	0.24
	2001-2008	0.11	0.11	7.9	0.008	1.1	79	0.08	2.2	158	0.16	2.8	202	0.20
	2009-2016	0.07	0.07	5.0	0.005	0.7	50	0.05	1.4	101	0.10	1.8	130	0.13
	2018-2020	0.06	0.06	8.6	0.009	0.6	36	0.04	2.4	173	0.17	1.5	108	0.11
Laundry room operator	1996-2000	-	-	-	-	-	-	-	-	-	-	-	-	-
	2001-2008	-	-	-	-	-	-	-	-	-	-	-	-	-
	2009-2016	-	-	-	-	-	-	-	-	-	-	-	-	-
	2018-2020	-	-	-	-	-	-	-	-	-	-	-	-	-
Table S2 continued…..														

			**Duration of exposure**											
			**1 year**			**10 years**			**20 years**			**25 years**		
Job	Time period	Mean f/ml	CE f/ml-years	Cases per/10^5	% Incid	CE f/ml-years	Cases per/10^5	% Incid	CE f/ml-years	Cases per/10^5	% Incid	CE f/ml-years	Cases per/10^5	% Incid
Pipe joints operators	1996-2000	0.13	0.13	9.4	0.01	1.3	94	0.09	2.6	187	0.19	3.3	238	0.24
	2001-2008	0.11	0.11	7.9	0.01	1.1	79	0.08	2.2	158	0.16	2.8	202	0.20
	2009-2016	0.05	0.05	3.6	0.005	0.5	36	0.04	1.0	72	0.07	1.3	94	0.09
	2018-2020	0.05	0.05	3.6	0.005	0.5	36	0.04	1.0	72	0.07	1.3	94	0.09
Fulllength pipe operator	1996-2000	0.13	0.13	9.4	0.01	1.3	94	0.09	2.6	187	0.19	3.3	238	0.24
	2001-2008	0.11	0.11	7.9	0.01	1.1	79	0.08	2.2	158	0.16	2.8	202	0.20
	2009-2016	0.07	0.07	5.0	0.01	0.7	50	0.05	1.4	101	0.10	1.8	130	0.13
	2018-2020	-	-	-	-	-	-	-	-	-	-	-	-	-
Multicutter operator	1996-2000	0.13	0.13	9.4	0.01	1.3	94	0.09	2.6	187	0.19	3.3	238	0.24
	2001-2008	0.12	0.12	8.6	0.01	1.2	86	0.09	2.4	173	0.17	3.0	216	0.22
	2009-2016	0.07	0.07	5.0	0.01	0.7	50	0.05	1.4	101	0.10	1.8	130	0.13
	2018-2020	0.04	0.04	2.9	0.003	0.4	29	003	0.8	58	0.06	1.0	72	0.07
Overall factory		0.12	0.12	8.6	0.009	1.2	86	0.09	2.2	158	0.16	3.0	216	0.22

% Incid – Percentage incidence; CE – Cumulative. Cases/10^5 – cancer mortality/morbidity cases per 100 000 workers exposed.

Table S3: Cumulative exposure on **mesothelioma** cancer risk by job, time period, exposure duration of 1, 10, 20 and 25 years: Harare factory

			**Duration of exposure**											
			**1 year**			**10 years**			**20 years**			**25 years**		
Job	Time period	Mean f/ml	CE f/ml-years	Cases per/10^5	% Incid	CE f/ml-years	Cases per/10^5	% Incid	CE f/ml-years	Cases per/10^5	% Incid	CE f/ml-years	Cases per/10^5	% Incid
Saw cutting operator	1996-2000	0.19	0.19	8.0	0.008	1.9	70	0.07	3.8	138	0.14	4.8	174	0.17
	2001-2008	0.13	0.13	5.8	0.006	1.3	48	0.05	2.6	95	0.10	3.3	120	0.12
	2009-2016	0.07	0.07	3.7	0.004	0.7	26	0.03	1.4	52	0.05	1.8	66	0.06
	2018-2020	0.10	0.10	4.8	0.005	1.0	37	0.04	2.0	73	0.07	2.5	91	0.09
Fettling table operator	1996-2000	0.12	0.12	5.5	0.006	1.2	44	0.04	2.4	88	0.09	3.0	109	0.11
	2001-2008	0.12	0.12	5.5	0.006	1.2	44	0.04	2.4	88	0.09	3.0	109	0.11
	2009-2016	-	-	-	-	-	-		-	-	-	-	-	-
	2018-2020	0.11	0.11	5.1	0.005	1.1	41	0.04	2.2	81	0.08	2.8	102	0.11
Moulded goods operator	1996-2000	0.11	0.11	5.1	0.005	1.1	41	0.04	2.2	81	0.08	2.8	102	0.11
	2001-2008	0.11	0.11	5.1	0.005	1.1	41	0.04	2.2	81	0.08	2.8	102	0.11
	2009-2016	0.05	0.05	3.0	0.003	0.5	19	0.02	1.0	37	0.04	1.3	48	0.09
	2018-2020	0.11	0.11	5.1	0.005	1.1	41	0.04	2.2	81	0.08	2.8	102	0.11
Kollergang operator	1996-2000	0.13	0.13	5.8	0.006	1.3	48	0.05	2.6	95	0.10	3.3	120	0.12
	2001-2008	0.12	0.12	5.5	0.006	1.2	44	0.04	2.4	88	0.09	3.0	109	0.11
	2009-2016	0.07	0.07	3.7	0.004	0.7	26	0.03	1.4	52	0.05	1.8	66	0.06
	2018-2020	0.12	0.12	5.5	0.004	1.2	44	0.04	2.4	88	0.09	3.0	109	0.11
Ground hard waste operator	1996-2000	0.16	0.16	6.9	0.007	1.6	59	0.06	3.2	117	0.12	3.3	120	0.12
	2001-2008	0.13	0.13	5.8	0.006	1.3	48	0.05	2.6	95	0.10	3.3	120	0.12
	2009-2016	0.07	0.07	3.7	0.004	0.7	26	0.03	1.4	52	0.05	1.8	66	0.07
	2018-2020	0.12	0.12	5.5	0.006	1.2	44	0.04	2.4	88	0.09	3.0	109	0.11
Laundry room operator	1996-2000	0.13	0.13	5.8	0.006	1.3	48	0.05	2.6	95	0.10	3.3	120	0.11
	2001-2008	0.13	0.13	5.8	0.006	1.3	48	0.05	2.6	95	0.10	3.3	120	0.11
	2009-2016	0.05	0.05	3.0	0.003	0.5	19	0.02	1.0	37	0.04	1.3	48	0.05
	2018-2020	0.11	0.11	5.1	0.005	1.1	41	0.04	2.2	81	0.08	2.8	102	0.11

Overall factory		0.11	0.11	5.1	0.005	1.1	41	0.04	2.2	81	0.08	2.8	102	0.10

% Incid – Percentage incidence; CE – Cumulative. Cases/10^5 – cancer mortality/morbidity cases per 100 000 workers exposed

Table S4: Cumulative exposure on Mesothelioma risk by job, time period, exposure duration of 1, 10, 20 and 25 years: Bulawayo factory

			**Duration of exposure**											
			**1 year**			**10 years**			**20 years**			**25 years**		
Job	Time period	Mean f/ml	CE f/ml-years	Cases per/10^5	% Incid	CE f/ml-years	Cases per/10^5	% Incid	CE f/ml-years	Cases per/10^5	% Incid	CE f/ml-years	Cases per/10^5	% Incid
Saw cutting operator	1996-2000	0.17	0.17	7.2	0.007	1.7	63	0.06	3.4	124	0.12	4.3	156	0.16
	2001-2008	0.12	0.12	5.4	0.005	1.2	44	0.04	2.4	88	0.09	3.0	109	0.11
	2009-2016	0.06	0.06	3.3	0.003	0.6	23	0.02	1.2	44	0.04	1.5	55	0.06
	2018-2020	0.05	0.05	3.0	0.003	0.5	19	0.02	1.0	37	0.04	1.3	48	0.05
Fettling table operator	1996-2000	0.17	0.17	7.2	0.007	1.7	63	0.06	3.4	124	0.12	4.3	156	0.16
	2001-2008	0.12	0.12	5.4	0.005	1.2	44	0.04	2.4	88	0.09	3.0	109	0.11
	2009-2016	-	-	-	-	-	-	-	-	-	-	-	-	-
	2018-2020	-	-	-	-	-	-	-	-	-	-	-	-	-
Moulded goods operator	1996-2000	-	-	-	-	-	-	-	-	-	-	-	-	-
	2001-2008	-	-	-	-	-	-	-	-	-	-	-	-	-
	2009-2016	-	-	-	-	-	-	-	-	-	-	-	-	-
	2018-2020	-	-	-	-	-	-	-	-	-	-	-	-	-
Kollergang operator	1996-2000	0.14	0.14	6.2	0.006	1.4	52	0.05	2.8	102	0.10	3.5	127	0.13
	2001-2008	0.12	0.12	5.4	0.005	1.2	44	0.04	2.4	88	0.09	3.0	109	0.11
	2009-2016	0.07	0.07	3.7	0.004	0.7	26	0.03	1.4	52	0.05	1.8	66	0.07
	2018-2020	0.06	0.06	3.3	0.003	0.6	23	0.02	1.2	44	0.04	1.5	55	0.06
Ground hard waste operator	1996-2000	0.13	0.13	5.8	0.006	1.3	48	0.05	2.6	95	0.10	3.3	120	0.12
	2001-2008	0.11	0.11	5.1	0.005	1.1	41	0.04	2.2	81	0.09	2.8	102	0.10
	2009-2016	0.07	0.07	3.7	0.004	0.7	26	0.03	1.4	52	0.05	1.8	66	0.07
	2018-2020	0.06	0.06	3.3	0.003	0.6	23	0.02	2.4	88	0.09	1.5	55	0.06

Laundry room operator	1996-2000	-	-	-	-	-	-	-	-	-	-	-	-	-
	2001-2008	-	-	-	-	-	-	-	-	-	-	-	-	-
	2009-2016	-	-	-	-	-	-	-	-	-	-	-	-	-
	2018-2020	-	-	-	-	-	-	-	-	-	-	-	-	-
Table S4 continued…..														
			**Duration of exposure**											
			**1 year**			**10 years**			**20 years**			**25 years**		
Job	Time period	Mean f/ml	CE f/ml-years	Cases per/10^5	% Incid	CE f/ml-years	Cases per/10^5	% Incid	CE f/ml-years	Cases per/10^5	% Incid	CE f/ml-years	Cases per/10^5	% Incid
Pipe joints operators	1996-2000	0.13	0.13	5.8	0.006	1.3	48	0.05	2.6	95	0.10	3.3	120	0.12
	2001-2008	0.11	0.11	5.1	0.005	1.1	41	0.04	2.2	81	0.08	2.8	102	0.11
	2009-2016	0.05	0.05	3.0	0.003	0.5	19	0.02	1.0	37	0.04	1.3	48	0.05
	2018-2020	0.05	0.05	3.0	0.003	0.5	19	0.02	1.0	37	0.04	1.3	48	0.05
Fulllength pipe operator	1996-2000	0.13	0.13	5.8	0.006	1.3	48	0.05	2.6	95	0.10	3.3	120	0.12
	2001-2008	0.11	0.11	5.1	0.005	1.1	41	0.04	2.2	81	0.08	2.8	102	0.10
	2009-2016	0.07	0.07	3.7	0.004	0.7	26	0.03	1.4	52	0.05	1.8	66	0.07
	2018-2020	-	-	-		-	-	-	-	-	-	-	-	-
Multicutter operator	1996-2000	0.13	0.13	5.8	0.006	1.3	48	0.05	2.6	95	0.10	3.3	120	0.12
	2001-2008	0.12	0.12	5.5	0.006	1.2	44	0.04	2.4	88	0.09	3.0	109	0.11
	2009-2016	0.07	0.07	3.7	0.004	0.7	26	0.03	1.4	52	0.05	1.8	66	0.07
	2018-2020	0.04	0.04	2.6	0.003	0.4	16	0.02	0.8	30	0.03	1.0	37	0.04
Overall factory		0.12	0.12	5.5	0006	1.2	44	0.04	2.2	81	0.08	3.0	109	0.11

% Incid – Percentage incidence; CE – Cumulative. Cases/10^5 – cancer mortality/morbidity cases per 100 000 workers exposed

Table S5: Cumulative exposure on gastrointestinal cancer risk by job, time period, exposure duration of 1, 10, 20 and 25 years: Harare factory

			**Duration of exposure**											
			**1 year**			**10 years**			**20 years**			**25 years**		
Job	Time period	Mean f/ml	CE f/ml-years	Cases per/10^5	% Incid	CE f/ml-years	Cases per/10^5	% Incid	CE f/ml-years	Cases per/10^5	% Incid	CE f/ml-years	Cases per/10^5	% Incid
Saw cutting operator	1996-2000	0.19	0.19	1.3	0.001	1.9	13.7	0.01	3.8	27.4	0.03	4.8	34.6	0.03
	2001-2008	0.13	0.13	0.9	0.001	1.3	9.3	0.009	2.6	18.7	0.02	3.3	23.8	0.02
	2009-2016	0.07	0.07	0.5	0.0005	0.7	5.0	0.005	1.4	10.1	0.01	1.8	12.9	0.01
	2018-2020	0.10	0.10	0.7	0.001	1.0	7.2	0.007	2.0	14.4	0.01	2.5	18.0	0.02
Fettling table operator	1996-2000	0.12	0.12	0.8	0.001	1.2	8.6	0.009	2.4	17.2	0.02	3.0	21.6	0.02
	2001-2008	0.12	0.12	0.8	0.001	1.2	8.6	0.009	2.4	17.2	0.02	3.0	21.6	0.02
	2009-2016	-	-	-	-	-	-	-	-	-	-	-	-	-
	2018-2020	0.11	0.11	0.8	0.001	1.1	7.9	0.008	2.2	15.8	0.02	2.8	20.2	0.02
Moulded goods operator	1996-2000	0.11	0.11	0.8	0.001	1.1	7.9	0.008	2.2	15.8	0.02	2.8	20.2	0.02
	2001-2008	0.11	0.11	0.8	0.001	1.1	7.9	0.008	2.2	15.8	0.02	2.8	20.2	0.02
	2009-2016	0.05	0.05	0.3	0.0003	0.5	3.6	0.004	1.0	7.2	0.01	1.3	9.4	0.01
	2018-2020	0.11	0.11	0.8	0.001	1.1	7.9	0.008	2.2	15.8	0.02	2.8	20.2	0.02
Kollergang operator	1996-2000	0.13	0.13	0.9	0.001	1.3	9.3	0.009	2.6	18.7	0.02	3.3	23.8	0.02
	2001-2008	0.12	0.12	0.8	0.001	1.2	8.6	0.009	2.4	17.2	0.02	3.0	21.6	0.02
	2009-2016	0.07	0.07	0.5	0.0005	0.7	5.0	0.005	1.4	10.1	0.01	1.8	12.9	0.01
	2018-2020	0.12	0.12	0.8	0.001	1.2	8.6	0.009	2.4	17.2	0.02	3.0	21.6	0.02
Ground hard waste operator	1996-2000	0.16	0.16	1.1	0.001	1.6	11.5	0.01	3.2	23.0	0.02	3.3	23.8	0.02
	2001-2008	0.13	0.13	0.9	0.001	1.3	9.3	0.009	2.6	18.7	0.02	3.3	23.8	0.02
	2009-2016	0.07	0.07	0.5	0.0005	0.7	5.0	0.005	1.4	10.1	0.01	1.8	12.9	0.01
	2018-2020	0.12	0.12	0.8	0.001	1.2	8.6	0.009	2.4	17.2	0.01	3.0	21.6	0.02

Laundry room operator	1996-2000	0.13	0.13	0.9	0.001	1.3	9.3	0.009	2.6	18.7	0.02	3.3	23.8	0.02
	2001-2008	0.13	0.13	0.9	0.001	1.3	9.3	0.009	2.6	18.7	0.02	3.3	23.8	0.02
	2009-2016	0.05	0.05	0.3	0.0003	0.5	3.6	0.004	1.0	7.2	0.01	1.3	9.3	0.01
	2018-2020	0.11	0.11	0.8	0.001	1.1	7.9	0.008	2.2	15.8	0.02	2.8	20.2	0.02
Overall factory		0.11	0.11	0.8	0.001	1.1	7.9	0.008	2.2	15.8	0.02	2.8	20.2	0.02

% Incid – Percentage incidence; CE – Cumulative. Cases/10^5 – cancer mortality/morbidity cases per 100 000 workers exposed

Table S6: Cumulative exposure on gastrointestinal cancer risk by job, time period, exposure duration of 1, 10, 20 and 25 years: Bulawayo factory

			**Duration of exposure**											
			**1 year**			**10 years**			**20 years**			**25 years**		
Job	Time period	Mean f/ml	CE f/ml-years	Cases per/10^5	% Incid	CE f/ml-years	Cases per/10^5	% Incid	CE f/ml-years	Cases per/10^5	% Incid	CE f/ml-years	Cases per/10^5	% Incid
Saw cutting operator	1996-2000	0.17	0.17	1.2	0.001	1.7	12.2	0.01	3.4	24.5	0.02	4.3	31.0	0.03
	2001-2008	0.12	0.12	0.8	0.001	1.2	8.6	0.009	2.4	17.3	0.02	3.0	21.6	0.02
	2009-2016	0.06	0.06	0.4	0.0004	0.6	4.3	0.004	1.2	8.6	0.009	1.5	10.8	0.01
	2018-2020	0.05	0.05	0.4	0.0004	0.5	3.6	0.004	1.0	7.2	0.007	1.3	9.3	0.01
Fettling table operator	1996-2000	0.17	0.17	1.2	0.001	1.7	12.2	0.01	3.4	24.5	0.02	4.3	31.0	0.03
	2001-2008	0.12	0.12	0.8	0.001	1.2	8.6	0.009	2.4	17.3	0.02	3.0	21.6	0.02
	2009-2016	-	-	-	-	-	-	-	-	-	-	-	-	-
	2018-2020	-	-	-	-	-	-	-	-	-	-	-	-	-
Moulded goods operator	1996-2000	-	-	-	-	-	-	-	-	-	-	-	-	-
	2001-2008	-	-	-	-	-	-	-	-	-	-	-	-	-
	2009-2016	-	-	-	-	-	-	-	-	-	-	-	-	-
	2018-2020	-	-	-	-	-	-	-	-	-	-	-	-	-
Kollergang operator	1996-2000	0.14	0.14	1.0	0.001	1.4	10.1	0.01	2.8	20.1	0.02	3.5	25.2	0.03
	2001-2008	0.12	0.12	0.8	0.001	1.2	8.6	0.009	2.4	17.3	0.02	3.0	21.6	0.02
	2009-2016	0.07	0.07	0.5	0.0005	0.7	5.0	0.005	1.4	10.1	0.01	1.8	12.9	0.01
	2018-2020	0.06	0.06	0.4	0.0004	0.6	4.3	0.004	1.2	8.6	0.009	1.5	10.8	0.01

Ground hard waste operator	1996-2000	0.13	0.13	0.9	0.001	1.3	9.3	0.009	2.6	18.7	0.02	3.3	23.8	0.02
	2001-2008	0.11	0.11	0.8	0.001	1.1	7.9	0.008	2.2	15.8	0.02	2.8	20.1	0.02
	2009-2016	0.07	0.07	0.5	0.0005	0.7	5.0	0.005	1.4	10.1	0.01	1.8	12.9	0.01
	2018-2020	0.06	0.06	0.4	0.0004	0.6	4.3	0.004	2.4	17.3	0.02	1.5	10.8	0.01
Laundry room operator	1996-2000	-	-	-	-	-	-	-	-	-	-	-	-	-
	2001-2008	-	-	-	-	-	-	-	-	-	-	-	-	-
	2009-2016	-	-	-	-	-	-	-	-	-	-	-	-	-
	2018-2020	-	-	-	-	-	-	-	-	-	-	-	-	-
Table S6 continued…..														
			**Duration of exposure**											
			**1 year**			**10 years**			**20 years**			**25 years**		
Job	Time period	Mean f/ml	CE f/ml-years	Cases per/10^5	% Incid	CE f/ml-years	Cases per/10^5	% Incid	CE f/ml-years	Cases per/10^5	% Incid	CE f/ml-years	Cases per/10^5	% Incid
Pipe joints operators	1996-2000	0.13	0.13	0.9	0.001	1.3	9.3	0.009	2.6	18.7	0.02	3.3	23.8	0.02.
	2001-2008	0.11	0.11	0.8	0.001	1.1	7.9	0.008	2.2	15.8	0.02	2.8	20.1	0.02
	2009-2016	0.05	0.05	0.3	0.0003	0.5	3.6	0.004	1.0	7.2	0.01	1.3	9.3	0.01
	2018-2020	0.05	0.05	0.3	0.0003	0.5	3.6	0.004	1.0	7.2	0.01	1.3	9.3	0.01
Fulllength pipe operator	1996-2000	0.13	0.13	0.9	0.001	1.3	9.3	0.009	2.6	18.7	0.02	3.3	23.8	0.02
	2001-2008	0.11	0.11	0.8	0.001	1.1	7.9	0.008	2.2	15.8	0.02	2.8	20.1	0.02
	2009-2016	0.07	0.07	0.5	0.0005	0.7	5.0	0.005	1.4	10.1	0.01	1.8	12.9	0.01
	2018-2020	-	-	-	-	-	-	-	-	-	-	-	-	-
Multicutter operator	1996-2000	0.13	0.13	0.9	0.001	1.3	9.3	0.009	2.6	18.7	0.02	3.3	23.8	0.02
	2001-2008	0.12	0.12	0.9	0.001	1.2	8.6	0.009	2.4	17.3	0.02	3.0	21.6	0.02
	2009-2016	0.07	0.07	0.5	0.0005	0.7	5.0	0.005	1.4	10.1	0.01	1.8	12.9	0.01
	2018-2020	0.04	0.04	0.3	0.0003	0.4	2.9	0.003	0.8	5.7	0.006	1.0	7.2	0.01
Overall factory		0.12	0.12	0.9	0.001	1.2	8.6	0.009	2.2	15.8	0.02	3.0	21.6	0.02

% Incid – Percentage incidence; CE – Cumulative. Cases/10^5 – cancer mortality/morbidity cases per 100 000 workers

Table S7: Overall summary estimates of cancer mortality cases by factory, job and duration of exposure of 1 year.

	**Harare factory**							**Bulawayo Factory**						
				Cancer cases per 100 000 exposed							Cancer cases per 100 000 exposed			
Job	N	Mean ±SD	CE	Lung	Meso	Gast	Tot	N	Mean ±SD	CE	Lung	Meso	Gast	Tot
Saw cutting operator	254	0.12±0.05	0.12	8.6	5.5	0.84	14.9	137	0.12±0.05	0.12	8.6	5.5	0.84	14.9
Fettling table operator	130	0.12±0.03	0.12	8.6	5.5	0.84	14.9	51	0.16±0.06	0.16	11.5	6.9	1.13	19.5
Moulded goods operator	197	0.10±0.04	0.10	7.2	4.8	0.70	12.7	-	-	-	-	-	-	-
Kollegang operator	212	0.10±0.04	0.10	7.2	4.8	0.70	12.7	126	0.10±0.04	0.10	7.2	4.8	0.70	12.7
Ground hard waste operator	176	0.12±0.04	0.12	8.6	5.5	0.84	14.9	76	0.11±0.04	0.11	7.9	5.1	0.77	13.8
Laundry operator	163	0.12±0.03	0.12	8.6	5.5	0.84	14.9	-	-	-	-	-	-	-
Pipe joints operator	-	-	-	-	-	-	-	103	0.11±0.03	0.11	7.9	5.1	0.77	13.8
Full length pipe operator	-	-	-	-	-	-	-	97	0.12±0.04	0.12	8.6	5.5	0.84	14.9
Multicutte r operator	-	-	-	-	-	-	-	66	0.12±0.03	0.12	8.6	5.5	0.84	14.9
Overall factory	1132	0.11±0.04	0.11	7.9	5.1	0.77	13.8	656	0.12±0.04	0.12	8.6	5.5	0.84	14.9

N—number of monthly-averaged personal chrysotile fibre concentrations, 1996—2020; SD—standard deviation; CE – Cumulative exposure in f/ml-years; Lung – lung cancer; Meso – Mesothelioma; Gast – Gastrointestinal cancer; Tot – Total estimated cancers at respective cumulative exposure

Table S8: Overall summary estimates of cancer mortality cases by factory, job and duration of exposure of 10 years.

	Harare Factory							Bulawayo Factory						
				Cancer cases per 100 000 exposed							Cancer cases per 100 000 exposed			
Job	N	Mean ±SD	CE	Lung	Mes	Gast	Tot	N	Mean ±SD	CE	Lung	Meso	Gast	Tot
Saw cutting operator	254	0.12±0.05	1.2	86.4	44	8.6	139	137	0.12±0.05	1.2	86.4	44	8.6	139
Fettling table operator	130	0.12±0.03	1.2	86.4	44	8.6	139	51	0.16±0.06	1.6	115	44	8.6	168
Moulded goods operator	197	0.10±0.04	1.0	72.0	37	7.2	116	-	-	-	-	-	-	-
Kollegang operator	212	0.10±0.04	1.0	72.0	37	7.2	116	126	0.10±0.04	1.0	72	37	7.2	116
Ground hard waste operator	176	0.12±0.04	1.2	86.4	44	8.6	139	76	0.11±0.04	1.1	79	41	7.9	128
Laundry operator	163	0.12±0.03	1.2	86.4	44	8.6	139	-	-	-	-	-	-	-
Pipe joints operator	-	-	-	-	-	-	-	103	0.11±0.03	1.1	79	41	7.9	128
Full length pipe operator	-	-	-	-	-	-	-	97	0.12±0.04	1.2	86.4	44	8.6	139
Multicutter operator	-	-	-	-	-	-	-	66	0.12±0.03	1.2	86.4	44	8.6	139
Overall factory	1132	0.11±0.04	1.1	79.2	40	7.9	127	656	0.12±0.04	1.2	86.4	44	8.6	139

N—number of monthly-averaged personal chrysotile fibre concentrations, 1996—2020; SD—standard deviation; CE – Cumulative exposure in f/ml-years; Lung – lung cancer; Meso – Mesothelioma; Gast – Gastrointestinal cancer; Tot – Total estimated cancers at respective cumulative exposure

Table S9: Overall summary estimates of cancer mortality cases by factory, job and duration of exposure of 20 years

	Harare Factory							Bulawayo Factory						
				Cancer cases per 100 000 exposed							Cancer cases per 100 000 exposed			
Job	N	Mean ±SD	CE	Lung	Mes	Gast	Tot	N	Mean ±SD	CE	Lung	Meso	Gast	Tot
Saw cutting operator	254	0.12±0.05	2.4	173	88	17	278	137	0.12±0.05	2.4	173	88	17	278
Fettling table operator	130	0.12±0.03	2.4	173	88	17	278	51	0.16±0.06	3.2	230	117	22	369
Moulded goods operator	197	0.10±0.04	2.0	144	73	14	231	-	-	-	-	-	-	-
Kollegang operator	212	0.10±0.04	2.0	144	73	14	231	126	0.10±0.04	2.0	144	73	14	231
Ground hard waste operator	176	0.12±0.04	2.4	173	88	17	278	76	0.11±0.04	2.2	158	81	16	255
Laundry operator	163	0.12±0.03	2.4	173	88	17	278	-	-	-	-	-	-	-
Pipe joints operator	-	-	-	-	-	-	-	103	0.11±0.03	2.2	158	81	16	255
Full length pipe operator	-	-	-	-	-	-	-	97	0.12±0.04	2.4	173	88	17	278
Multicutte r operator	-	-	-	-	-	-	-	66	0.12±0.03	2.4	173	88	17	278
Overall factory	1132	0.11±0.04	2.2	158	81	16	255	656	0.12±0.04	2.4	173	88	17	278

N—number of monthly-averaged personal chrysotile fibre concentrations, 1996—2020; SD—standard deviation; CE – Cumulative exposure in f/ml-years; Lung – lung cancer; Meso – Mesothelioma; Gast – Gastrointestinal cancer; Tot – Total estimated cancers at respective cumulative exposur

Table S10: Overall summary estimates of cancer mortality cases by factory, job and duration of exposure of 25 years

	Harare Factory							Bulawayo Factory						
				Cancer cases per 100 000 exposed							Cancer cases per 100 000 exposed			
Job	N	Mean ±SD	CE	Lung	Mes	Gast	Tot	N	Mean ±SD	CE	Lung	Meso	Gast	Tot
Saw cutting operator	254	0.12±0.05	3.0	216	109	22	347	137	0.12±0.05	3.0	216	109	22	347
Fettling table operator	130	0.12±0.03	3.0	216	109	22	347	51	0.16±0.06	4.0	288	146	29	463
Moulded goods operator	197	0.10±0.04	2.5	180	91	18	289	-	-	-	-	-	-	-
Kollegang operator	212	0.10±0.04	2.5	180	91	18	289	126	0.10±0.04	2.5	180	91	18	289
Ground hard waste operator	176	0.12±0.04	3.0	216	109	22	347	76	0.11±0.04	2.8	203	102	20	325
Laundry operator	163	0.12±0.03	3.0	216	109	22	347	-	-	-	-	-	-	-
Pipe joints operator	-	-	-	-	-	-	-	103	0.11±0.03	2.8	203	102	20	325
Full length pipe operator	-	-	-	-	-	-	-	97	0.12±0.04	3.0	216	109	22	347
Multicutte r operator	-	-	-	-	-	-	-	66	0.12±0.03	3.0	216	109	22	347
Overall factory	1132	0.11±0.04	2.8	203	81	20	304	656	0.12±0.04	3.0	216	109	22	347

N—number of monthly-averaged personal chrysotile fibre concentrations, 1996—2020; SD—standard deviation; CE – Cumulative exposure in f/ml-years; Lung – lung cancer; Meso – Mesothelioma; Gast – Gastrointestinal cancer; Tot – Total estimated cancers at respective cumulative exposure.

Table S11: Estimates of asbestosis incidence after 25 years of exposure

	Harare factory					Bulawayo factory				
Job	N	Mean ±SD	CE	% Incidence	Cases per 100 000	N	Mean ±SD	CE	% Incidence	Cases per 100 000
Saw cutting operator	254	0.12±0.05	3.0	0.16	160	137	0.12±0.05	3.0	0.16	160
Fettling table operator	130	0.12±0.03	3.0	0.16	160	51	0.16±0.06	4.0	0.22	220
Moulded goods operator	197	0.10±0.04	2.5	0.14	140	-	-	-	-	-
Kollergang operator	212	0.10±0.04	2.5	0.14	140	126	0.10±0.04	2.5	0.14	140
Ground hard waste operator	176	0.12±0.04	3.0	0.16	160	76	0.11±0.04	2.8	0.15	150
Laundry room operator	163	0.12±0.03	3.0	0.16	160	-	-	-	-	-
Pipe joints operator	-	-	-	-	-	103	0.11±0.04	2.8	0.15	150
Full length pipe operator						97	0.12±0.04	3.0	0.16	160
Multicutter operator						66	0.12±0.04	3.0	0.16	160
Overall factory	1132	0.11±0.04	2.8	0.15	150	656	0.12±0.03	3.0	0.16	160

N—number of monthly-averaged personal chrysotile fibre concentrations, 1996—2020; SD—standard deviation; CE – Cumulative exposure in f/ml-years.

Table S12: Estimated number of workers working at various jobs in the chrysotile asbestos cement manufacturing factories

		**Harare factory**		**Bulawayo**	
Job	Time period	No. of workers by time period	Maximum possible workers per job	No. of workers by time period	Maximum workers per job
Saw cutting operator	1996-2000<br>2001-2008<br>2009-2016<br>2018-2020	54<br>45<br>27<br>3	54	54<br>54<br>54<br>54	54
Fettling table operator	1996-2000<br>2001-2008<br>2009-2016<br>2018-2020	15<br>15<br>15<br>-	15	15<br>15<br>15<br>15	15
Moulded Goods operator	1996-2000<br>2001-2008<br>2009-2016<br>2018-2020	18<br>18<br>18<br>-	18	-<br>-<br>-<br>-	-
Kollergang operator	1996-2000<br>2001-2008<br>2009-2016<br>2018-2020	12<br>12<br>12<br>-	12	12<br>12<br>12<br>12	12
Ground hard waste operator	1996-2000<br>2001-2008<br>2009-2016<br>2018-2020	6<br>6<br>6<br>-	6	6<br>6<br>6<br>6	6
Laundry room operator	1996-2000<br>2001-2008<br>2009-2016<br>2018-2020	2<br>2<br>2<br>-	2	-<br>-<br>-<br>-	-
Pipe joints operators	1996-2000<br>2001-2008<br>2009-2016<br>2018-2020	-<br>-<br>-<br>-	-	27<br>27<br>27<br>27	27
Fulllength pipe operator	1996-2000<br>2001-2008<br>2009-2016<br>2018-2020	-<br>-<br>-<br>-	-	27<br>27<br>27<br>27	27
Multi-cutter operator	1996-2000<br>2001-2008<br>2009-2016<br>2018-2020	-<br>-<br>-<br>-	-	12<br>12<br>12<br>12	12
Total Average			107		153
